# Different Infectivity of Swine Enteric Coronaviruses in Cells of Various Species

**DOI:** 10.3390/pathogens13020174

**Published:** 2024-02-15

**Authors:** Zhongyuan Li, Yunyan Chen, Liang Li, Mei Xue, Li Feng

**Affiliations:** State Key Laboratory for Animal Disease Control and Prevention, Harbin Veterinary Research Institute, Chinese Academy of Agricultural Sciences, Harbin 150069, China

**Keywords:** TGEV, PEDV, PDCoV, replication, aminopeptidase N (APN)

## Abstract

Swine enteric coronaviruses (SECoVs), including porcine deltacoronavirus (PDCoV), transmissible gastroenteritis virus (TGEV), porcine epidemic diarrhea virus (PEDV), and swine acute diarrhea syndrome coronavirus (SADS-CoV), have caused high mortality in piglets and, therefore, pose serious threats to the pork industry. Coronaviruses exhibit a trend of interspecies transmission, and understanding the host range of SECoVs is crucial for improving our ability to predict and control future epidemics. Here, the replication of PDCoV, TGEV, and PEDV in cells from different host species was compared by measuring viral genomic RNA transcription and protein synthesis. We demonstrated that PDCoV had a higher efficiency in infecting human lung adenocarcinoma cells (A549), Madin–Darby bovine kidney cells (MDBK), Madin–Darby canine kidney cells (MDCK), and chicken embryonic fibroblast cells (DF-1) than PEDV and TGEV. Moreover, trypsin can enhance the infectivity of PDCoV to MDCK cells that are nonsusceptible to TGEV. Additionally, structural analyses of the receptor ectodomain indicate that PDCoV S1 engages Aminopeptidase N (APN) via domain II, which is highly conserved among animal species of different vertebrates. Our findings provide a basis for understanding the interspecies transmission potential of these three porcine coronaviruses.

## 1. Introduction

Coronaviruses (CoVs) are members of the *Nidovirales* order, family *Coronaviridae*, subfamily *Orthocoronaviridae*, which are enveloped viruses with a positive-sense, single-stranded RNA genome [1]. CoVs have a wide range of natural hosts, infecting humans, pigs, birds, and other vertebrates, and exhibit a propensity for interspecies transmission. According to serological and genomic methods, there are four genera in the subfamily of *Orthocoronaviridae*, including Alphacoronavirus, Betacoronavirus, Gammacoronavirus, and Deltacoronavirus [2]. They cause respiratory and digestive tract infections in humans and animals. The Alphacoronaviruses, transmissible gastroenteritis virus (TGEV), porcine epidemic diarrhea virus (PEDV), swine acute diarrhea syndrome coronavirus (SADS-CoV) and the Deltacoronavirus porcine deltacoronavirus (PDCoV) have caused a high number of deaths among piglets, posing serious threats to the pork industry [3,4]. TGEV was first reported and isolated in 1946 in the United States. The prevalence of TGEV was then reported successively in many countries in Europe and Asia, and the disease was first discovered in China in 1958 [5]. PEDV was first isolated and discovered in Europe about 40 years ago and has been endemic in Eurasia since the 1990s [6]. PEDV broke out in China around 2010 [7]. Compared to the discovery of PEDV, the first report of PDCoV was relatively new and was detected in 2012 during molecular surveillance of coronavirus in avian and mammal species in Hong Kong [8]. SADS-CoV is an HKU2-related bat coronavirus, which has spread from Rhinolophus bats to pigs, leading to a large-scale outbreak of severe diarrhea in piglets in China [9,10]. Except for domestic pigs, the wild boars are reservoirs for enteric swine CoVs. Previous studies have confirmed the presence of PEDV and TGEV coinfection in wild boars in the Campania region [11].

Coronaviruses have a diameter of 80–120 nm and are spherical or oval in shape [12]. The main structural proteins of coronaviruses are spike (S) protein, membrane (M) protein, envelope (E) protein, and nucleocapsid (N) protein. The S protein forms the coronal processes of most coronaviruses, while the surface protuberances of certain specific coronaviruses (such as HCoV-HKU1) are made of dimer hemagglutinin-esterase (HE) proteins [13]. By attaching to host cell receptors, the S protein primarily facilitates virus invasion and determines viral tissue or host tropism. The M protein is the primary component of the envelope, which has three transmembrane domains [14]. The E protein and M protein maintain the structure and size of the viral envelope [15]. The N protein binds to the viral genomic RNA and packages it into the virion [16].

The capacity of the virus to attach and effectively employ a receptor within a different host is what determines whether cross-species transmission will be successful, making the S protein the primary factor in CoV emergence. The initial step in coronavirus infection is the binding of viral S protein to host cell surface receptors. Similar to other viruses with capsular membranes, SeCoVs also mediated the virus to complete the adsorption and invasion of cells through the S protein. In the host cell, S protein is cleaved into S1 and S2 parts under the action of protease, S1 can bind to the receptor on the surface of the host cell, and the main role of S2 subunit is to mediate virus–cell and cell–membrane fusion. First, S1 binds to the receptor. The N-terminal domain (NTD) of S1 binds to sugars, the C-terminal domain (CTD) binds to cellular protein receptors, and the fusion peptide (FP) of the S2 subunit is exposed by the enzyme. After FP fuses with the cell membrane, two heptad repeats (HR) of S2 subunit interact with each other to form a stable fusion center [17]. When the fusion protein folds backwards, the viral envelope fuses with the cell membrane and completes the virus entry process [18].

Aminopeptidase N (APN), also known as CD13, is a 150 kDa type II glycoprotein that belongs to the family of membrane-bound metalloproteinases [19]. APN is mainly expressed on the surface of epithelial cells of the small intestine, respiratory tract, and kidney. Through the S protein, CoVs can recognize amino acids specific to the host APN and infect the host. It can be cleaved by trypsin into two subunits, the NTD (95 KDa) and the CTD (50 KDa) [20]. APN was found to be the cellular infection receptor for some coronaviruses, including human coronavirus 229E (HCoV-229E), feline infectious peritonitis virus (FIPV), canine coronavirus (CCoV), and TGEV [21,22,23,24]. The 36–223 aa, 349–591 aa, and 592–963 aa of porcine APN (pAPN) may be the three main regions that bind to the TGEV S protein [25]. Previous studies have shown that PEDV can be successfully infected by overexpressing pAPN in MDCK cells, preliminarily identifying the functional receptor of PEDV is pAPN [26]. Later studies proved that transgenic mouse models expressing pAPN were sensitive to PEDV [27]. However, recent research has reported PEDV can still infect pAPN knockout pigs [28]. PDCoV can infect the pAPN knocked-down swine testis cells (ST), suggesting that there are still other cell surface proteins in pigs that act as receptors for PEDV and PDCoV [28]. Besides, several other coronavirus receptors have been identified. Recent research indicates that SARS-CoV-2 binds to DPP4/CD26 when it enters respiratory tract cells [29]. Moreover, some researchers demonstrated that SARS-CoV-2 uses the SARS-CoV receptor ACE2 for entry and the serine protease TMPRSS2 for S protein priming [30]. ASGR1 and KREMEN1, as entry receptors of SARS-CoV-2, play an important role in SARS-CoV-2 infection independent of that of ACE2 [31]. Transferrin receptor (TfR) has been proven to be another receptor that affects the entry of SARS-CoV-2 into the host [32].

SADS-CoV can infect cell lines originating from various species, including A549, Vero, ST, IPEC-J2, BHK-21, DF-1 cells, etc. [33]. In the present study, a set of experiments was carried out to compare the susceptibility of different readily available cell lines to PEDV, TGEV and PDCoV infection. This result provides a basis for understanding the interspecies transmission potential of these three porcine coronaviruses.

## 2. Materials and Methods

### 2.1. Cells, Viruses and Antibodies

Human lung adenocarcinoma cells (A549), swine intestinal epithelial cell line (IPI-2I), Baby hamster kidney cell line (BHK-21), African green monkey kidney cells (Vero E6), Madin–Darby bovine kidney cells (MDBK), Madin–Darby canine kidney cells (MDCK), and chicken embryonic fibroblast cells (DF-1) were stored by Harbin Veterinary Research Institute, Chinese Academy of Agricultural Sciences. IPI-2I, Vero E6, BHK-21, MDBK, MDCK, and DF-1 cells were maintained in Dulbecco’s Modified Eagle Medium (DMEM, Gibco, Carlsbad, CA, USA) supplemented with 10% fatal bovine serum (FBS, Gibco, Carlsbad, CA, USA) and antibiotics (100 μg/mL streptomycin and 100 U/mL penicillin). A549 cells were maintained in the DMEM–nutrient mixture F-12 (Ham) (1:1) (DMEM-F12) (Gibco, Carlsbad, CA, USA) supplemented with 10% FBS (Gibco, Carlsbad, CA, USA). All of the above cells were cultured in a humidified atmosphere at 37 °C and 5% CO_2_. TGEV Hua strain H87 was propagated from virulent strain H16 (GenBank accession number: FJ755618) by sequential passaging in PK-15 cells in our laboratory [34]. PEDV G2 strain LNCT2 (GenBank accession number: KT323980) was isolated in Vero E6 cells in our laboratory [35]. The PDCoV NH strain (GenBank accession number: KU981062.1) was isolated and preserved in our laboratory [36]. TGEV nucleocapsid (N) protein monoclonal antibody (MAb), PEDV S protein MAb, and PDCoV S Protein Polyclonal Antibody (PAb) were prepared and stored in our laboratory.

### 2.2. Virus Infection

Cells of different species were seeded onto 24-well plates at a density of 4 × 10^5^/well in a complete culture medium incubated for 24 h. Cells were infected with TGEV, PEDV and PDCoV (MOI = 0.02, 0.2 and 1) or mock infected with DMEM. After 2 h of infection at 37 °C, unbound viruses were removed by washing the cells three times with DMEM. Then, cells were cultured in maintenance medium (DMEM supplemented with 0.1% trypsin) at 37 °C. Cells were harvested at 2 hpi or 24 hpi.

### 2.3. RNA Isolation and Quantitative PCR Analysis

Cells were infected with TGEV, PEDV, and PDCoV at an MOI of 0.02, 0.2 and 1 and collected at 24 hpi. RNA was extracted using Simply P total RNA extraction kit (BioFlux, Beijing, China) according to the manufacturer’s instructions in 24-well plates. Total RNA (2 μg) was reverse transcribed into complementary DNA (cDNA) using M-MLV reverse transcriptase (Takara, Otsu, Shiga, Japan) and an Oligo(dT)_15_ primer (Takara, Otsu, Shiga, Japan). The cDNA was subsequently subjected to real-time quantitative PCR (qPCR) in triplicate by using FastStart Essential DNA Green Master (Roche, Basel, Switzerland). Primers based on the TGEV N gene, PEDV N and PDCoV N gene were synthesized for quantification of the virus genome in qPCR. The primers are shown in Table 1 and were created using the program Oligo 6. The results were analyzed based on the cycle threshold (ΔΔCT) method using Glyceraldehyde-3-phosphate dehydrogenase (GAPDH) as the internal reference for the relevant viral replication levels.

### 2.4. Immunofluorescence Assay (IFA)

Cells from human, bovine, canine, avian, hamster and monkey were seeded in 96-well plates. When they reached 90% confluency, cells were infected with TGEV, PEDV, and PDCoV at an MOI of 1 for 24 h, washed three times with PBS and fixed with 4% paraformaldehyde for 30 min at 4 °C. The cells were then washed three times with PBST, and 0.2% Triton X-100 (T8787, Sigma, St. Louis, MO, USA) was added for 20 min at room temperature. Fixed cells were blocked using 5% skim milk (232100, BD Difco, Sparks, MD, USA) in PBS for 1 h at 37 °C. The cells were then incubated with primary antibodies (MAb against TGEV-N, PEDV-S and PAb against PDCoV-S prepared in our laboratory) for 2 h at 37 °C, washed three times with PBST, and then incubated with the Alexa Fluor 546 goat anti-mouse IgG (A11001, ThermoFisher Scientific, Waltham, MA, USA) for 1 h at 37 °C. The nuclei were visualized with 1 μg/mL of DAPI (D9542, Sigma, St. Louis, MO, USA) for 10 min at room temperature. After cells were washed three times with PBST, images were acquired with an EVOS FL Cell Imaging System fluorescence microscope (ThermoFisher Scientific, Waltham, MA, USA) at 10× and 20× magnification. The percentage of infected cells was calculated by counting the number of infected cells in a 10× microscopic field of view.

### 2.5. Comparison of Homology of APN between Different Species

The APN sequences of different species were downloaded from the NCBI website. The sequences obtained were aligned and analyzed by using DNAstar software (v7.1, DNASTAR Inc., Madison, WI, USA).

### 2.6. Statistical Analysis

All results shown in the figures were analyzed using GraphPad Prism 9 and expressed as mean ± standard deviation (SD). Comparisons among all groups were examined using *t*-test. Differences were considered statistically significant if the *p* value < 0.05. The significance level (*p* value) was set at <0.05 (*), <0.01 (**), and <0.001 (***).

## 3. Results

### 3.1. A549 Cells Are Permissive for PDCoV and PEDV 

**Figure 1:**
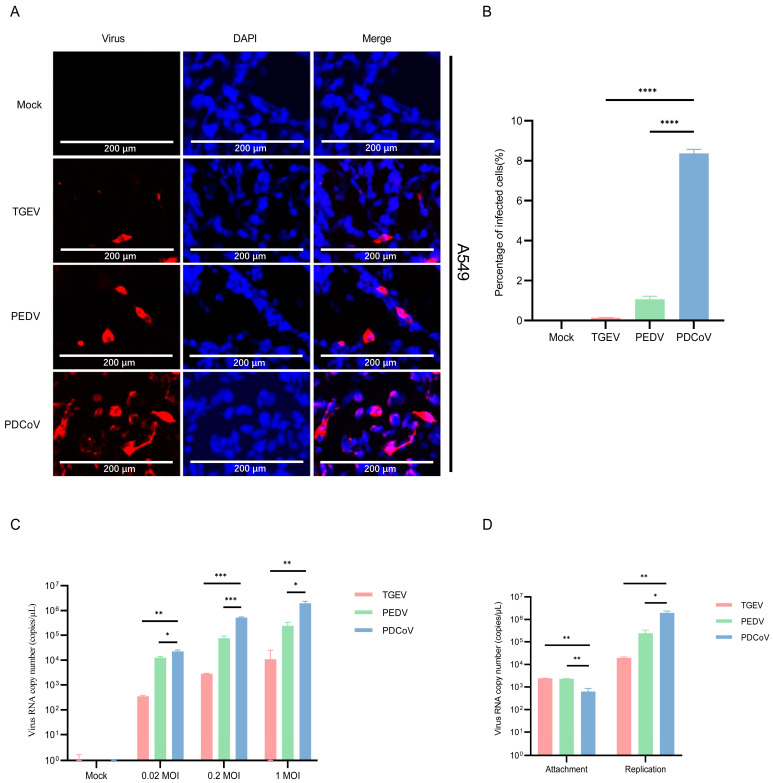
Infection of PDCoV, PEDV and TGEV in human A549 cells. (**A**) A549 cells were infected with TGEV, PEDV, and PDCoV (MOI = 1) for 24 h, and the virus infection was analyzed by IFA. (**B**) The percentage of infected cells was determined by the formula (the number of infected cells/the number of total cells) × 100. (**C**) A549 cells were infected with TGEV, PEDV, and PDCoV (MOI = 0.02, 0.2 and 1) for 24 h, cells were lysed and detected by qPCR. Means and SD of the results from three independent experiments are shown. (**D**) A549 cells were infected with TGEV, PEDV, and PDCoV (MOI = 1) for 2 h or 24 h, cells were lysed and detected by qPCR. *, *p* < 0.05.**, *p* < 0.01. ***, *p* < 0.001. ****, *p* < 0.0001. The *p* value was calculated using *t*-test.

Here, we evaluated the susceptibility of A549 cells to SECoVs. To this end, the A549 cells were infected with TGEV, PEDV, and PDCoV (MOI = 1) for 24 h, and the infection levels were assessed by IFA. At 24 h post-infection (hpi), we observed higher fluorescence in A549 cells infected with PDCoV compared with that observed in cells infected with PEDV and TGEV (Figure 1A). Almost no fluorescent cells were detected in A549 cells infected with TGEV (Figure 1B). Next, we observed the replication of PDCoV, PEDV and TGEV in a multiplicity of infections (MOI) of 0.02, 0.2 or 1 by qPCR. The results showed a dose-dependent increase in virus RNA copy number (Figure 1C). Comparing the RNA copy number of the virus at 24 hpi with the post-adsorption stage, and the results showed that the replication efficiency of PDCoV in A549 cells is higher than that of PEDV and TGEV (Figure 1D). These results indicate that human A549 cells are more effective in supporting PDCoV replication compared to PEDV and TGEV.

### 3.2. MDBK Cells Are Permissive for PDCoV, PEDV and TGEV

**Figure 2:**
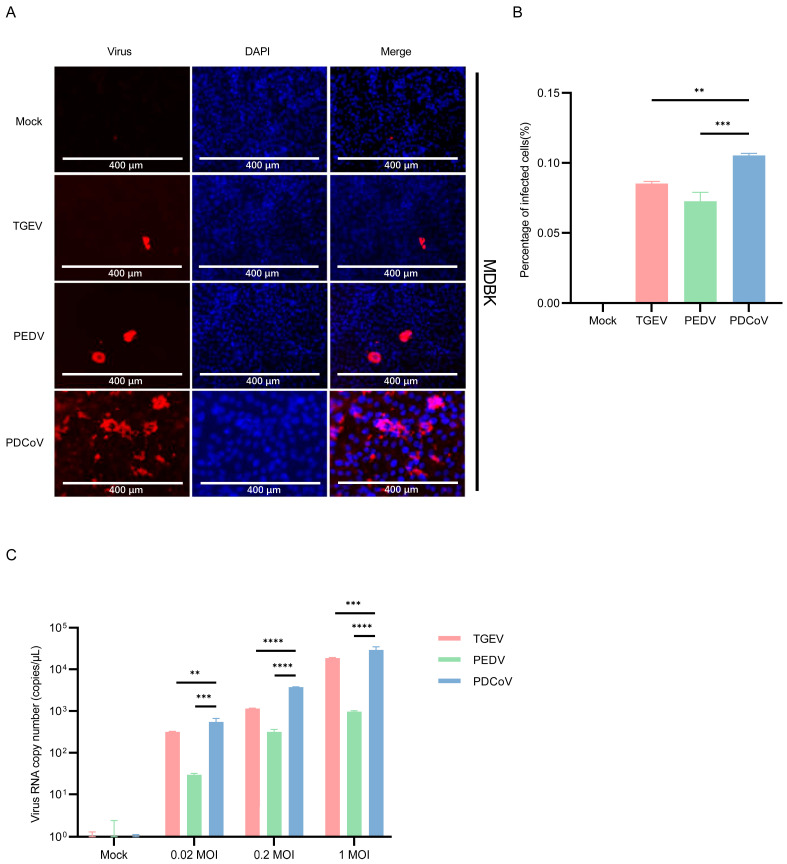
Infection of PDCoV, PEDV and TGEV in MDBK cells. (**A**) MDBK cells were infected with TGEV, PEDV, and PDCoV (MOI = 1) for 24 h, and the virus infection was analyzed by IFA. (**B**) The percentage of infected cells was determined by the formula (the number of infected cells/the number of total cells) × 100. (**C**) MDBK cells were infected with TGEV, PEDV, and PDCoV (MOI = 0.02, 0.2 and 1) for 24 h, cells were lysed and detected by qPCR. Means and SD of the results from three independent experiments are shown. **, *p* < 0.01. ***, *p* < 0.001. ****, *p* < 0.0001. The *p* value was calculated using *t*-test.

To determine whether different organs of bovine origin were susceptible to infection with enteric coronavirus, the MDBK cells, a cell line derived from a kidney of an apparently normal adult steer, were used. The results of immunofluorescence showed that TGEV, PEDV, and PDCoV could infect MDBK cells, and higher PDCoV antigens in the cells compared with PEDV and TGEV (Figure 2A,B). In addition, mean PDCoV RNA titers in PDCoV-inoculated MDBK cells were higher than PEDV and TGEV (Figure 2C). 

### 3.3. PDCoV Had a Higher Efficiency in Infecting MDCK Than PEDV and TGEV

**Figure 3:**
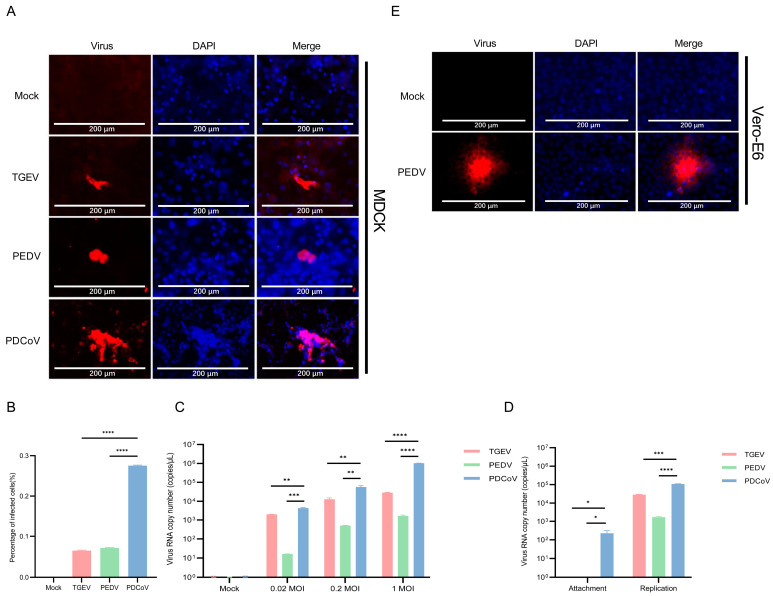
Infection of PDCoV, PEDV and TGEV in MDCK cells. (**A**) MDCK cells were infected with TGEV, PEDV, and PDCoV (MOI = 1) for 24 h, and the virus infection was analyzed by IFA. (**B**) The percentage of infected cells was determined by the formula (the number of infected cells/the number of total cells) × 100. (**C**) MDCK cells were infected with TGEV, PEDV, and PDCoV (MOI = 0.02, 0.2 and 1) for 24 h, cells were lysed and detected by qPCR. Means and SD of the results from three independent experiments are shown. (**D**) MDCK cells were infected with TGEV, PEDV, and PDCoV (MOI = 1) for 2 h or 24 h, cells were lysed and detected by qPCR. (**E**) Vero-E6 cells were infected with the supernatant of PEDV-infected MDCK cells, and the virus infection was analyzed by IFA. *, *p* < 0.05. **, *p* < 0.01. ***, *p* < 0.001. ****, *p* < 0.0001. The *p* value was calculated using *t*-test.

To understand whether MDCK cells are susceptible to SECoVs, immunofluorescence staining was performed in PDCoV, TGEV and PEDV-infected cells. The results showed that only a few cells were infected when 0.1% trypsin was added, with an infection rate of approximately 0.3% (Figure 3B). The fluorescence number in PDCoV-infected MDCK cells is significantly higher than that of PEDV and TGEV (Figure 3A). Then, viral copies were determined by RT-qPCR. As shown in Figure 3C, the viral copies of PDCoV were higher than those of PEDV and TGEV. Comparing the RNA copy number of the virus at 24 hpi with the post-adsorption stage, and the results showed that the replication efficiency of PDCoV in MDCK cells is higher than that of PEDV and TGEV (Figure 3D). 

Since the percentage of PEDV-infected MDCK cells are quite low, it is necessary to verify whether infectious progeny viruses could be produced, or they were abortive infections. So, we performed subsequent rounds of infections. Firstly, MDCK cells were infected with the supernatant of PEDV-infected MDCK cells, and IFA assay showed no specific fluorescence. Then, Vero-E6 cells were infected with the supernatant of PEDV-infected MDCK cells, and IFA assay showed a small amount of specific fluorescence (Figure 3E). These results indicated that although the percentage of infected cells was low, infectious progeny viruses could be produced.

**Figure 4:**
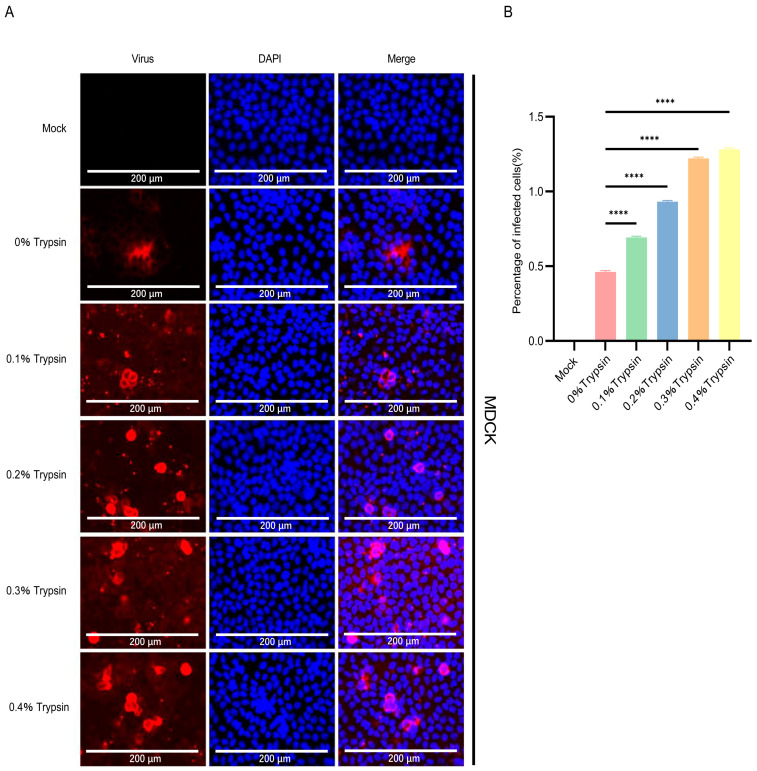
Trypsin prompts the replication of PDCoV in MDCK cells. (**A**) PDCoV (MOI = 1) was incubated with MDCK cells for 24 h in the absence or presence of different doses of trypsin, washed three times with PBS and analyzed by IFA. (**B**) The percentage of infected cells was determined by the formula (the number of infected cells/the number of total cells) × 100. Means and SD of the results from three independent experiments are shown. ****, *p* < 0.0001. The *p* value was calculated using *t*-test.

We evaluated whether higher doses of trypsin can help PDCoV infect MDCK cells. To this end, different concentrations of trypsin (0.1%, 0.2%, 0.3%, and 0.4%) were added to PDCoV to infect MDCK cells, and the replication of PDCoV was detected by IFA. As the amount of trypsin added gradually increases, the replication of PDCoV in MDCK cells increases in a dose-dependent manner (Figure 4A,B). These results indicate that trypsin significantly prompts the replication during PDCoV infection of MDCK cells.

### 3.4. DF-1 Cells Are More Susceptible to PDCoV

**Figure 5:**
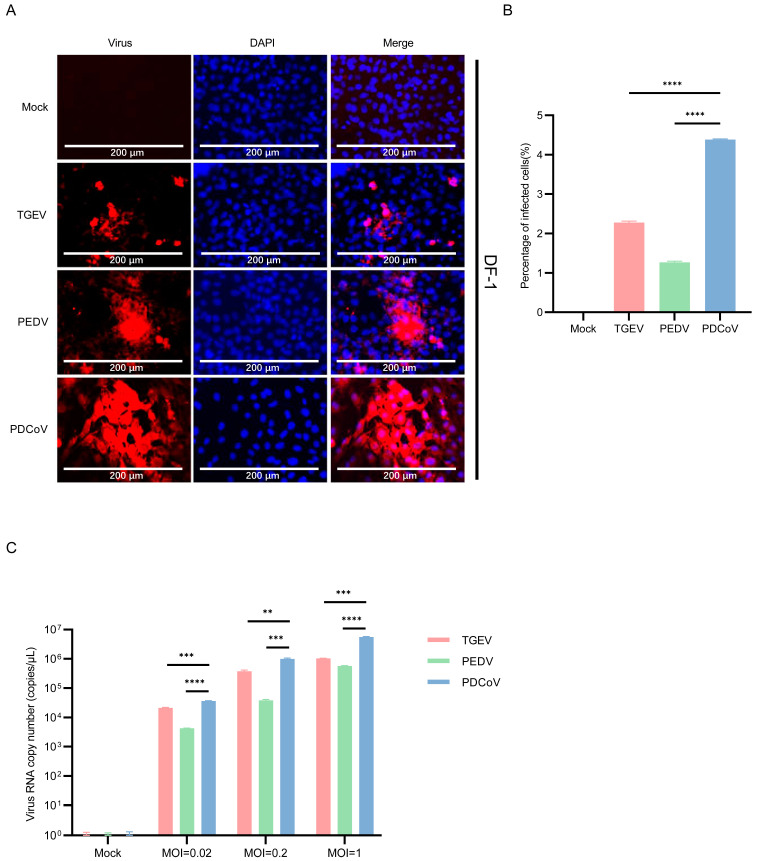
Infection of PDCoV, PEDV and TGEV in DF-1 cells. (**A**) DF-1 cells were infected with TGEV, PEDV, and PDCoV (MOI = 1) for 24 h, and the virus infection was analyzed by IFA. (**B**) The percentage of infected cells was determined by the formula (the number of infected cells/the number of total cells) × 100. (**C**) DF-1 cells were infected with TGEV, PEDV, and PDCoV (MOI = 0.02, 0.2 and 1) for 24 h, cells were lysed and detected by qPCR. Means and SD of the results from three independent experiments are shown. **, *p* < 0.01. ***, *p* < 0.001. ****, *p* < 0.0001. The *p* value was calculated using *t*-test.

Previously, it was demonstrated that PDCoV is susceptible to infection in DF-1 cells [37]. To compare the infection efficiency of DF-1 to SECoVs, TGEV, PEDV, and PDCoV were infected and then evaluated virus replication by immunostaining at 24 hpi. The results showed that the replication of PDCoV in DF-1 cells was significantly higher than that of PEDV and TGEV (Figure 5A,B). The mean copy number of PDCoV RNA in DF-1 cells inoculated with PDCoV was higher than that in PEDV and TGEV (Figure 5C). The observations collectively reveal that PDCoV replicates better in human, bovine, canine, and avian cells compared to alphacoronavirus TGEV and PEDV.

### 3.5. Vero E6 and BHK-21 Cells Are Both More Susceptible to PEDV

**Figure 6:**
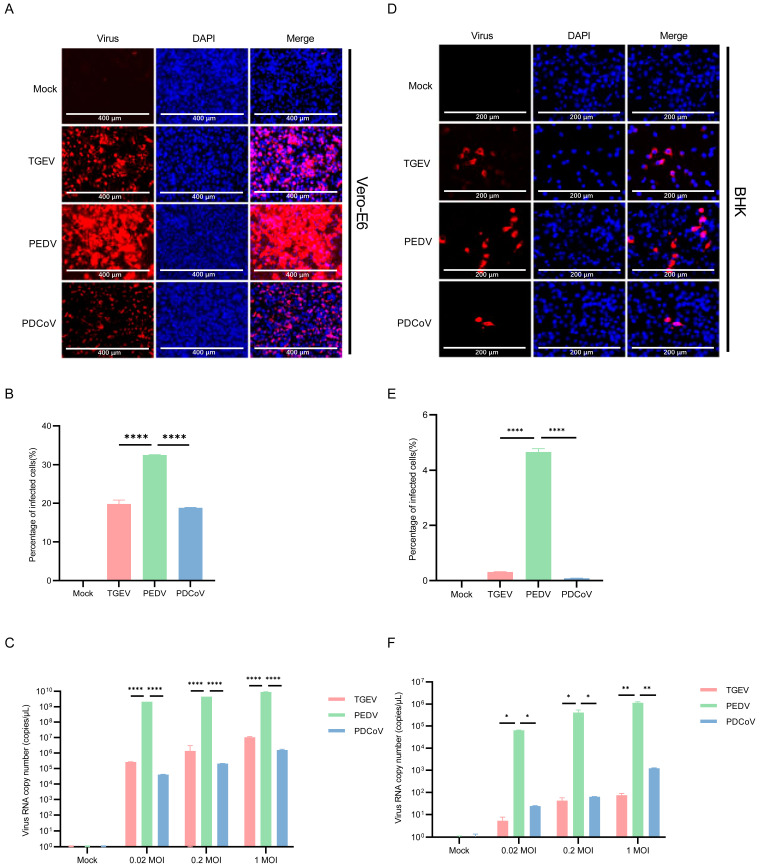
Infection of PDCoV, PEDV and TGEV in Vero-E6 and BHK-21 cells. (**A**,**D**) Vero-E6 and BHK-21 cells were infected with TGEV, PEDV, and PDCoV (MOI = 1) for 24 h, and the virus infection was analyzed by IFA. (**B**,**E**) The percentage of infected cells was determined by the formula (the number of infected cells/the number of total cells) × 100. (**C**,**F**) Vero-E6 and BHK-21 cells were infected with TGEV, PEDV, and PDCoV (MOI = 0.02, 0.2 and 1) for 24 h, cells were lysed and detected by qPCR. Means and SD of the results from three independent experiments are shown. *, *p* < 0.05. **, *p* < 0.01. ****, *p* < 0.0001. The *p* value was calculated using *t*-test.

It was previously reported that PEDV could be continuously propagated in Vero E6 cells, a monkey cell line that does not express pAPN [38]. The immunofluorescence staining and qPCR results showed that PEDV maintained a considerably greater number of infected cells and viral load than PDCoV and TGEV (Figure 6A–C). The number of PEDV-infected cells reached about 30% (Figure 6B). Then, we selected BHK-21 cells for TGEV, PEDV, and PDCoV inoculation to see the difference in virus replication between mice-derived cells and pig-derived cells. We inoculated the BHK-21 cells with PDCoV, TGEV, and PEDV (MOI = 0.02, 0.2, and 1), followed by immunofluorescence staining and qPCR at 24 hpi. The results of immunofluorescence and qPCR showed that TGEV, PEDV, and PDCoV can effectively infect BHK-21 cells (Figure 6D–F), and PEDV has the best replication effect.

### 3.6. IPI-2I Cells Are More Susceptible to TGEV

**Figure 7:**
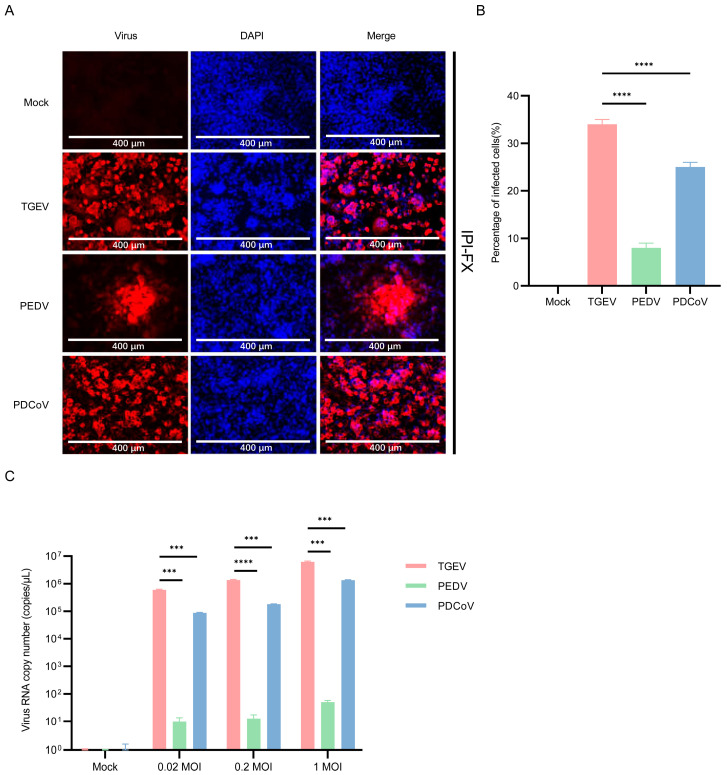
Infection of PDCoV, PEDV and TGEV in IPI-2I cells. (**A**) IPI-2I cells were infected with TGEV, PEDV, and PDCoV (MOI = 1) for 24 h, and the virus infection was analyzed by IFA. (**B**) The percentage of infected cells was determined by the formula (the number of infected cells/the number of total cells) × 100. (**C**) IPI-2I cells were infected with TGEV, PEDV, and PDCoV (MOI = 0.02, 0.2 and 1) for 24 h, cells were lysed and detected by qPCR. Means and SD of the results from three independent experiments are shown. ***, *p* < 0.001. ****, *p* < 0.0001. The *p* value was calculated using *t*-test.

To test the susceptibility of IPI-2I cells to different SECoVs, IPI-2I cells were infected with TGEV, PEDV, and PDCoV. The results of immunofluorescence showed that IPI-2I cells are highly susceptible to TGEV and PDCoV infection, but only a small number of PEDV-positive cells could be detected (Figure 7A). The number of virus-infected cells reached about 35% for TGEV, 25% for PDCoV and 8% for PEDV (Figure 7B). Next, the genomic RNA of SECoVs in IPI-2I cells was detected by qPCR, and the results showed that the mean copy number of TGEV RNA in IPI-2I cells was higher than that in PDCoV and PEDV (Figure 7C). These results suggested that IPI-2I cells are highly susceptible to TGEV infection.

### 3.7. Similarity Analysis of pAPN with Other Species

**Figure 8:**
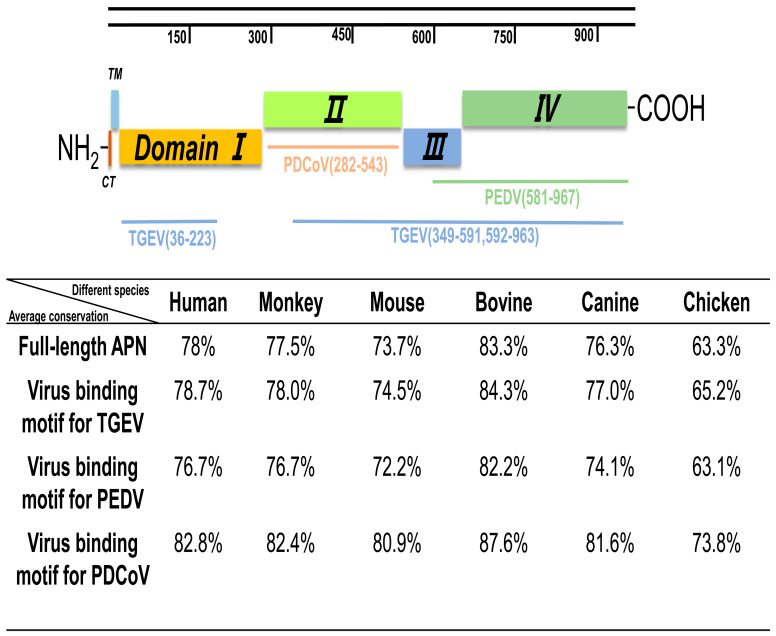
Schematic representation of the APN protein with the different domains and virus–binding regions indicated. Virus–binding regions of TGEV, PEDV, and PDCoV are indicated, as previously reported [23,26,41,42]. The porcine APN amino acid sequence comparison with human, monkey, mouse, canine, bovine and chicken shows a homology range from 63.3% to 83.3%. When the virus-binding motif was compared, domain II is highly conserved across animal species of the different vertebrate classes.

Cross-species transmission by coronaviruses is foremost determined by the virus’ ability to bind receptors of new hosts. TGEV primarily utilizes pAPN as a receptor, while PEDV and PDCoV not only utilizes pAPN, but it can also utilize other unknown receptors in the absence of pAPN [39]. Structural analyses of the pAPN ectodomain have revealed four independently folded domains, termed domains I through IV [40], with PEDV S1 binding to domain III and IV and PDCoV S1 engaging pAPN via domain II, whereas TGEV S1 binds to part of domains I and II, and the full regions of domains III and IV [37]. Among these four domains, domain II is highly conserved among animal species of different vertebrates (Figure 8). The chicken APN had a 73.8% amino acid identity to pAPN in domain II, which is the lowest among different species (Figure 8).

## 4. Discussion

Although intestinal epithelial cells are the primary target of PEDV, TGEV and PDCoV infection, the spectrum of cell types that are infected in vitro is diverse. The comparison of PDCoV, TGEV and PEDV infection in cells from various host species identifies the varied responses of different cell types to SECoVs. Here we found that PDCoV had a higher efficiency in infecting A549 cells, MDBK, MDCK, and DF-1 than PEDV and TGEV. Furthermore, structural analyses of the receptor ectodomain indicate that PDCoV S1 engages APN via domain II, which is more conservative than TGEV and PEDV engaging APN. This help us understand the zoonotic potential of porcine coronaviruses.

It has been previously reported that human hepatoma (Huh7) cells were susceptible to PDCoV infection [37]. The adenocarcinoma human alveolar basal epithelial cell line A549 has been widely used for in vitro studies of influenza A virus replication [43]. This study is the first to show that the A549 cell line is susceptible to infection by PDCoV and PDCoV transcription and protein synthesis takes place in A549 cells. Several reports showed that PDCoV RNA could be detected in low to moderate quantities in lung and multiple intestinal tissues in mice and chickens, indicating multisystemic viral distribution occurred during PDCoV infection [44,45,46]. Previous studies confirmed that PEDV caused intestinal infection by intranasal inoculation [47]. It will be important to establish whether cells in the respiratory tract can be infected by PDCoV and the alternative route of PDCoV transmission.

PEDV is more susceptible to Vero-E6 and BHK-21 cells at a higher level than that of TGEV and PDCoV. The growth potential of coronavirus in a variety of cells correlated with the utilization of its receptor molecule [37,42]. Most known coronaviruses become infective in cells when their functional receptor molecule is expressed by transfection with a plasmid encoding the receptor molecule [37]. This indicates that Vero cells and BHK-21 cells susceptible to PEDV infection express functional receptors. Further studies are needed to investigate the potential receptors in intestine infected with PEDV.

A previous study has shown that primary bovine mesenchymal cells are susceptible to infection with PDCoV and PEDV [48]. Based on the data from MDBK, bovine kidney cells were susceptible to infection with PDCoV, which is consistent with the previously reported susceptibility of gnotobiotic calves to PDCoV infection [49]. The previous study showed that gnotobiotic calves infected with PDCoV develop acute subclinical infections, with no histological damage in the intestine and other major organs (including the kidneys and heart), and no PDCoV antigen detected. However, fecal virus RNA continues to shed and serum transforms [49]. On the other hand, gnotobiotic calves infected with PEDV did not exhibit fecal virus shedding, serum transformation, histopathology, or detectable PEDV antigens in the corresponding tissues. Our results of immunofluorescence showed lower PEDV antigens in MDBK cells compared with PDCoV (Figure 2A), which may explain its inability to infect in calves.

It has been reported that MDCK cells were not susceptible to PEDV [50]. Although PDCoV can infect MDCK cells, it cannot replicate extensively in MDCK cells. The amino acid comparison of different species of APN shows that the homology between bovine APN and pAPN, canine APN and pAPN is higher than that of chicken APN and pAPN (Figure 8). Interestingly, immunofluorescence assays showed that the susceptibility of PDCoV to DF-1 cells was significantly higher than that of MDBK and MDCK cells. Although PDCoV can attach to and enter cells lacking APN, it fails to initiate efficient replication in them [51]. The results of this study highlighted that there may be other key receptors that assist PDCoV in infecting cells of different species, resulting in a wide host spectrum.

PEDV and TGEV also replicated in cell lines from different animal species, such as chicken (DF-1), humans (A549) and monkeys (Vero-E6). However, PEDV and TGEV have been found only within the pig population [52]. The main target cells of SECoVs in vivo are intestinal villous epithelial cells. However, the susceptibility of pig IPI-2I intestinal epithelial cells to the three SECoVs is completely different. We found that IPI-2I cells are highly sensitive to TGEV, but not to PEDV. Similarly, the influenza virus replicates well in MDCK cells, but there is no factual evidence to suggest that it can be transmitted from dog to dog. These observations support the discrepant in vivo and in vitro results from the previous studies [48,49].

Oncolytic viruses (OVs) have made significant progress in cancer treatment, and OVs are becoming increasingly effective anti-cancer drugs. Previously, adenoviruses, herpesviruses, reovirus, and paramyxovirus have been shown to have oncolytic properties [53,54,55,56]. In 2009, the oncolytic activity of coronavirus mouse hepatitis virus (MHV) was first confirmed in vivo [57]. Recent research indicates that in some cases, SARS-CoV-2 infection can trigger oncolytic effects and anti-tumor immune responses [58]. Whether the SECoVs have oncolytic potential deserves further exploration.

## 5. Conclusions

In conclusion, the results indicate that PDCoV can infect cells from different host species in vitro and undergo mRNA transcription and protein synthesis, especially in DF-1 cells from poultry and A549 cells from humans, with replication efficiency much higher than TGEV and PEDV. Interestingly, although SECoVs can infect cells of different species, there have been no reports of cross-species transmission of TGEV and PEDV, except for PDCoV. This may be due to the very low infection efficiency of TGEV and PEDV on cells of other species, as well as the presence of some immune responses in vivo, which deserves further in-depth research.

## Figures and Tables

**Table 1:** Specific primers and probe for qPCR.

Gene Name	Primer Sequences 5′-3′
TGEV-N-F	GCAGGTAAAGGTGATGTGACAAG
TGEV-N-R	GACACAGATGGAACACATTCAGC
PEDV-N-F	GCAGTAATTCCTCAGATCCTC
PEDV-N-R	GTAGTGTCAGATGCAATGAG
PDCoV-N-F	AGCAACCACTCGTGTTACTTG
PDCoV-N-R	CAACTCTGAAACCTTGAGCTG
GAPDH-R	CCTTCCGTGTCCCTACTGCCAAC
GAPDH-F	GACGCCTGCTTCACCACCTTCT

## Data Availability

All relevant data are included within the paper.
